# Transmitted antiretroviral drug resistance mutations in newly diagnosed HIV-1 positive patients in Turkey

**DOI:** 10.7448/IAS.17.4.19750

**Published:** 2014-11-02

**Authors:** Murat Sayan, Fatma Sargýn, Dilara Inan, Dilek Yýldýz Sevgi, Aysel Kocagül Celikbas, Kadriye Yasar, Figen Kaptan, Selda Sayýn Kutlu, Nuriye Tasdelen Fýsgýn, Ayse Inci, Nurgül Ceran, Ýlkay Karaoðlan, Atahan Cagatay, Mustafa Kemal Celen, Suda Tekin Koruk, Bahadýr Ceylan, Taner Yýldýrmak, Halis Akalýn, Volkan Korten, Ayse Willke

**Affiliations:** 1 Clinical Laboratory, Kocaeli University, Kocaeli, Turkey; 2 Infectious Diseases, Goztepe Hospital, Medeniyet University, Istanbul, Turkey; 3 Infectious Diseases, Akdeniz University, Antalya, Turkey; 4 Infectious Diseases, Sisli Etfal Hospital, Istanbul, Turkey; 5 Infectious Diseases, Ankara Numune Hospital, Ankara, Turkey; 6 Infectious Diseases, Bakýrköy Egitim Arastýrma Hospital, Istanbul, Turkey; 7 Infectious Diseases, Atatürk Hospital, Katip Celebi University, Izmir, Turkey; 8 Infectious Diseases, Pamukkale University, Denizli, Turkey; 9 Infectious Diseases, Mayis University, Samsun, Turkey; 10 Infectious Diseases, Istanbul Kanuni Hospital, Istanbul, Turkey; 11 Infectious Diseases, Haydarpasa Educational and Research Hospital, Istanbul, Turkey; 12 Infectious Diseases, Gaziantep University, Gaziantep, Turkey; 13 Infectious Diseases, Istanbul University, Istanbul, Turkey; 14 Infectious Diseases, Dicle University, Diyarbakir, Turkey; 15 Infectious Diseases, Harran University, Urfa, Turkey; 16 Infectious Diseases, Bezm-i Alem University, Istanbul, Turkey; 17 Infectious Diseases, Istanbul Okmeydani Hospital, Istanbul, Turkey; 18 Infectious Diseases, Uludag University, Bursa, Turkey; 19 Infectious Diseases, Marmara University, Istanbul, Turkey; 20 Infectious Diseases, Kocaeli University, Kocaeli, Turkey

## Abstract

**Introduction:**

The objective of this study was to determine the transmitted drug resistance mutations (TDRMs) in newly diagnosed HIV-1 positive patients in Turkey.

**Materials and Methods:**

The study was carried out between 2009 and 2014 and antiretroviral naïve 774 HIV-1 infected patients from 19 Infectious Diseases and Clinical Microbiology Departments in Turkey were included; gender: 664 (86%) male, median age: 37 (range; 1–77), median CD4+T-cell: 360 (range; 1–1320) count/mm^3^, median HIV-RNA load: 2.10+E6 (range; 4.2+E2–7.41+E8) IU/mL. HIV-1 drug resistance mutations were detected by population based sequencing of the reverse transcriptase (codon 41–238) and protease (codon 1–99) domains of pol gene of HIV-1, and analyzed according to the criteria by the World Health Organization 2009 list of surveillance drug resistance mutations [1].

**Results:**

The patients had TDRMs to NRTIs (K65R, M184V), NNRTIs (K101E, K103N/S, G190A/E/S, Y181I/C, Y188H/L) and PIs (M46L, I54V, L76V, V82L/T, N83D, I84V, L90M). The prevalence of overall TDRMs was 6.7% (52/774). Resistance mutations were found to be 0.7% (6/774), 4.1% (32/774) and 2.1% (17/774) to NRTIs, NNRTIs and PIs drug groups, respectively. Three patients had NRTIs+NNRTs resistance mutations (M184V+K103N) as multi-class drug resistance. However, thymidine analogue resistance mutations (TAMs) determined two distinct genotypic profiles in the HIV-1 reverse transcriptase: TAM1: M41L, L210W and T215Y, and TAM2: D67N, K70R, K219E/Q, and T215F. The prevalence of TAM1 and TAM2 were 7.7% (60/774) and 4.3% (34/774), respectively.

**Conclusions:**

The TDRMs prevalence of antiretroviral naïve HIV-1 infected patients may be suggested current situation of Turkey. These long-term and large-scale results show that the resistance testing must be an integral part of the management of HIV infection in Turkey.
